# A STAT3-decoy oligonucleotide induces cell death in a human colorectal carcinoma cell line by blocking nuclear transfer of STAT3 and STAT3-bound NF-κB

**DOI:** 10.1186/1471-2121-12-14

**Published:** 2011-04-12

**Authors:** Inès Souissi, Imen Najjar, Laurent Ah-Koon, Pierre Olivier Schischmanoff, Denis Lesage, Stéphanie Le Coquil, Claudine Roger, Isabelle Dusanter-Fourt, Nadine Varin-Blank, An Cao, Valeri Metelev, Fanny Baran-Marszak, Remi Fagard

**Affiliations:** 1 INSERM, Unité 978, Bobigny, France; 2 Université Paris 13, UFR SMBH, Bobigny, France; 3 AP-HP, Hôpital Avicenne, Service de biochimie, Bobigny, France; 4 AP-HP, Hôpital Avicenne, Service d'hématologie biologique, Bobigny, France; 5 Moscow State University, Moscow, Russia; 6 Institut Cochin, Université Paris Descartes, CNRS (UMR 8104), Paris, France; 7 INSERM, Unité 1016, Paris, France; 8 Groupe de Vectorisation, UFR SMBH, Université Paris 13, Bobigny, France

## Abstract

**Background:**

The transcription factor STAT3 (signal transducer and activator of transcription 3) is frequently activated in tumor cells. Activated STAT3 forms homodimers, or heterodimers with other TFs such as NF-κB, which becomes activated. Cytoplasmic STAT3 dimers are activated by tyrosine phosphorylation; they interact with importins via a nuclear localization signal (NLS) one of which is located within the DNA-binding domain formed by the dimer. In the nucleus, STAT3 regulates target gene expression by binding a consensus sequence within the promoter. STAT3-specific decoy oligonucleotides (STAT3-decoy ODN) that contain this consensus sequence inhibit the transcriptional activity of STAT3, leading to cell death; however, their mechanism of action is unclear.

**Results:**

The mechanism of action of a STAT3-decoy ODN was analyzed in the colon carcinoma cell line SW 480. These cells' dependence on activated STAT3 was verified by showing that cell death is induced by STAT3-specific siRNAs or Stattic. STAT3-decoy ODN was shown to bind activated STAT3 within the cytoplasm, and to prevent its translocation to the nucleus, as well as that of STAT3-associated NF-κB, but it did not prevent the nuclear transfer of STAT3 with mutations in its DNA-binding domain. The complex formed by STAT3 and the STAT3-decoy ODN did not associate with importin, while STAT3 alone was found to co-immunoprecipitate with importin. Leptomycin B and vanadate both trap STAT3 in the nucleus. They were found here to oppose the cytoplasmic trapping of STAT3 by the STAT3-decoy ODN. Control decoys consisting of either a mutated STAT3-decoy ODN or a NF-κB-specific decoy ODN had no effect on STAT3 nuclear translocation. Finally, blockage of STAT3 nuclear transfer correlated with the induction of SW 480 cell death.

**Conclusions:**

The inhibition of STAT3 by a STAT3-decoy ODN, leading to cell death, involves the entrapment of activated STAT3 dimers in the cytoplasm. A mechanism is suggested whereby this entrapment is due to STAT3-decoy ODN's inhibition of active STAT3/importin interaction. These observations point to the high potential of STAT3-decoy ODN as a reagent and to STAT3 nucleo-cytoplasmic shuttling in tumor cells as a potential target for effective anti-cancer compounds.

## Background

STAT3 belongs to the signal transducers and activators of transcription (STATs) family of transcription factors (TFs) [1]. STAT3 is activated in response to several cytokines and growth factors, including IL-6, epidermal growth factor (EGF), and interferon (IFN) α; STAT3 is also weakly activated in response to other cytokines, including IFNγ. Activation of STAT3 results from the phosphorylation of tyrosine 705, mediated by Janus Kinases (JAK), which are associated to cytokine receptors, and also by the Src and Abelson (Abl) families of protein tyrosine kinases [2]. STAT3 is also phosphorylated on serine 727, sometimes resulting in its activation. Following phosphorylation, STAT3 dimerizes and enters the nucleus by interacting with nuclear import proteins [3] of the karyopherin/importin family [4]. The importins interact with nuclear localization signals (NLS), one of which is located within the DNA binding domain (DBD) of STAT3 and is thought to be the most efficient [3,5]. Once in the nucleus, STAT3 activates the transcription of its target genes, including cyclin D1, survivin, VEGF, c-myc, Bcl-xL, and Bcl2 (see [6] for review). Once released from its DNA targets, STAT3 is dephosphorylated in the nucleus [7] and exported to the cytoplasm by a CRM1-dependent process [8]. STAT3 has been described as a key regulator of cell survival and proliferation [9]; its constitutive activation has been observed in many human tumors, including colon, breast, lung, pancreas and prostate cancers, melanoma, head and neck squamous carcinoma, multiple myeloma, mantle cell lymphoma, and glioma [10,11]. In addition, substituting amino acids located at the STAT3 dimer interface for cysteines yielded a stabilized STAT3 dimer that was able to induce a pseudotransformed phenotype [12]. Thus, its constitutive activation in tumor cells points to STAT3 as a valuable target for attacking tumor cells. Furthermore, despite its essential role in development [13], STAT3 is not essential for the functioning of mature cells [14]. Some STAT3 inhibitors are not specific, such as curcumin [15]. In contrast, Stattic, which prevents STAT3 dimerization by specifically interacting with its SH2 domain [16], is highly specific, and efficiently induces tumor cell death [16,17]. Despite its frequent involvement in cancer, which makes it a highly valuable target for inducing tumor cell death, STAT3 still lacks more specific inhibitors. Besides the SH2 domain, another potential target for highly selective STAT3 inhibitors is its DBD, since it selectively recognizes and binds DNA motifs in target genes. Decoy oligonucleotides (decoy ODNs) containing the TFs' DNA binding consensus sequences selectively inhibit them by binding to the DBD [18]. They can induce, *in vitro*, the death of tumor cells whose growth depends on these TFs [19]. This has notably been shown for several TFs, including NF-κB [20,21] and STAT3 [17,22-24]. STAT3-decoy ODN efficiently induced cell death in mouse xenografts of a head and neck squamous cell carcinoma [25]. One limitation of STAT3-decoy ODN is that despite the different functions of STAT1 and STAT3 in the cell, they recognize very similar DNA targets [26], with the result that STAT3-decoy ODN can inhibit either one or the other. For example, in the colon carcinoma cell line SW 480, the constitutive activation of STAT3 contributes to cell survival; its inhibition by STAT3-decoy ODN induces cell death. However, the ODN also blocks IFNγ-mediated cell death through STAT1 activation in the same cell line [17]. The actual mechanism through which decoy ODNs inhibit TFs is still unclear. Of the many studies demonstrating decoy ODN-mediated inhibition of TFs such as E2F, NF-κB [27], CRE and AP1 [28], none have specifically investigated the subcellular localization required for decoy ODNs to exercise their inhibitory action. A study on AP1 suggested that nuclear entry is required for decoy ODNs to inhibit targeted TFs [29]. Another study showed that a decoy ODN engineered to contain a nuclear localization signal (NLS) could enter the nucleus and efficiently inhibit p53 [30]. It is not clear yet whether these requirements depend on cellular systems or on the TFs that are targeted, since other studies have found that decoy ODNs did not have to enter the nucleus to exert their inhibitory effect [17,21]. In order to assess their possible use in human cancer, it will be important to understand the mechanism through which the decoy ODNs interfere with TFs and to determine whether nucleo-cytoplasmic shuttling is impaired. In the case of STAT3, constitutive shuttling from cytoplasm to nucleus has been demonstrated [8,31]. Furthermore, STAT3's localization seems to be predominantly nuclear [32], indicating that the shuttling mechanism could be a promising target for achieving effective STAT3 inhibition, as previously suggested [33]. Decoy ODNs' mechanism of action on STAT3 was therefore studied to determine whether nucleo-cytoplasmic shuttling was impaired, leading to STAT3 inhibition. Finally, since STAT3 has been reported to interact and synergize with NF-κB [34] in tumor cells [35], this study also addresses the functional interplay of NF-κB and decoy ODN.

## Methods

### Cell culture and reagents

SW 480 (colon adenocarcinoma) and MCF-7 (breast cancer) cell lines were grown in DMEM (GibcoBRL, Life technologies, Cergy-Pontoise, France), supplemented with 10% FCS (Lonza, Levallois-Perret, France) 100 U/mL penicillin, 10 μg/mL streptomycin (GibcoBRL), 1 mM sodium pyruvate (GibcoBRL), MEM vitamins 100 × (GibcoBRL) and 5 μg/mL plasmocin (Cayla InvivoGen, Toulouse, France). The KG-1 cells were grown in 10% FCS supplemented IMDM medium (GibcoBRL). For the STAT3 overexpression experiments the plasmid PLZst3α was used. The STAT3 DNA binding domain (DBD)-mutant containing two mutations in the DBD that completely prevented DNA binding but allowed dimerization and nuclear entry, was a kind gift from Dr. C. Horvath (Northwestern University, Chicago, USA) [36]. For some experiments, cells were treated with TNFα (20 ng/ml) (Sigma-Aldrich, Montigny le Bretonneux, France). To enhance STAT3 activation, cells were treated for 1 hr with IL-6 (50 ng/ml) (Sigma). Sodium orthovanadate (100 μM) (stock solution: 100 mM) was from Fischer (Illkirch, France), leptomycin B (LMB) (10 ng/ml) was from Sigma-Aldrich.

### RNA silencing

For cell infection with lentiviral shRNA, a set of two STAT1-targeting shRNAs that has previously been found to reduce the expression of STAT1 [37] was used and transduced as previously described [37]. Efficiency of infection was verified by measuring GFP by flow cytometry, and the efficacy of the inhibition of the shRNA's inhibition of STAT1 expression was verified by western blotting using a STAT1-specific antibody (Cell Signaling, Ozyme, St Quentin Fallavier, France).

For siRNA STAT3 silencing, the following double stranded siRNA oligonucleotide, previously shown to suppress STAT3 expression in a colorectal cell line [38], was purchased from Sigma-Aldrich: 5'-AACAUCUGCCUAGAUCGGCUAdTdT-3'; 3'-dTdTGUAGACGGAUCUAGCCGAU-5', along with a universal control set of siRNA (Sigma Aldrich). Cells (10^5 ^cells/well; density: 60%) were transfected using polyethylene imine (PEI) with 10 nM siRNA in culture medium without antibiotics. After 48 h or 72 h, cells were harvested and analyzed for annexin V binding by flow cytometry. In control cells, the irrelevant control siRNA was used. All experiments were performed in triplicate.

### Preparation of subcellular fractions

Cells (20 × 10^6^) were resuspended in cell lysis buffer containing 20 mM Hepes pH 7.4, 1 mM MgCl_2_, 10 mM KCl, 0.3% NP40, 0.5 mM DTT, 0.1 mM EDTA and protease inhibitors (CompeteTM, Boerhinger, France), and placed at 4°C for 5 min. The lysates were centrifuged at 14000 g for 5 min at 4°C, and the supernatant containing the cytoplasmic fraction was stored in aliquots at -80°C. The pellets were resuspended in cell lysis buffer adjusted to 20% glycerol and 0.35 M NaCl and placed at 4°C for 30 min. After centrifugation at 14000 g for 5 min at 4°C, the supernatant, containing the nuclear proteins, was stored at -80°C. Protein amounts were determined before use with the micro-BCA protein determination kit (Pierce, Perbio, Brebières, France).

### Decoy oligonucleotides

The STAT3-decoy ODNs used were: RHN(CH_2_)_6_- CATTTCCCGTAAATCGAAGATTTACGGGAAATG -(CH_2_)_3_NHR (hp STAT3-decoy ODN), derived from the serum-inducible element of the human c-fos promoter [39], and RHN(CH_2_)_6_- CATTTGCCACAATCGAAGATTGTGGCAAATG -(CH_2_)_3_NHR (hairpin STAT3-decoy mutated ODN) (Sigma-Proligo) where R was either H, FITC or biotin. The decoy NF-κB-ODN consisted of: RNH(CH_2_)_6_-CTGGAAAGTCCCTCGAAGAGGGACTTTCCAG-(CH_2_)_3_NHR (hairpin decoy NF-κB-ODN) and RHN(CH_2_)_6_-TGCAGTCACTACGCGAAGCGTAGTGACTGCA-(CH_2_)_3_NHR (hairpin scrambled decoy NF-κB-ODN) where R is either H or biotin. The synthesis of decoy oligonucleotides with R = H has been published elsewhere [17]. For biotin addition, 7-10 nanomoles of the oligodeoxynucleotide bearing 3'- and 5'-aminoalkyl linkers were dissolved in 20 μL of 0.1 M NaHCO_3_. EZ-Link NHS-biotin (Pierce, Rockford, USA) (10 μL of a 65 mM solution in dimethyl sulfoxide) was added, and the mixture was incubated at room temperature for 6-16 h in the dark. Then 25 μL of water were added, and the modified oligodeoxynucleotide was separated from the excess of hydrolyzed reagent by two consecutive separations on Micro Bio-Spin 6 columns following the manufacturer's recommendations. After the second spin, the biotinylated oligodeoxynucleotide was precipitated with ethanol-sodium acetate. In control experiments the previously published decoy NF-κB-ODN [21] was used. In some cases FITC-labeled or biotinylated decoy ODNs were obtained from Sigma-Aldrich. Note that the oligonucleotides used for cell death induction, pull-down assays and whole-cell pull-down assays were similar and could be used interchangeably, except that for pull-down biotinylated oligonucleotides had to be used.

### Preparation of liposomes

Liposomes were formulated using a cationic lipid (3β-[N-(N',N',N'-triethylaminopropane)-carbamoyl] cholesterol) iodide (TEAPC-Chol) and neutral colipid dioleoyl phosphatidylethanolamine (DOPE), as previously described [40]. The concentration of cationic lipid was monitored by UV spectroscopy at 226 nm and the value was used to calculate the charge ratio assuming one positive charge for each cationic lipid molecule.

### Gel electrophoresis, western blotting

Cells were washed in PBS, lysed in sample buffer (50 mM Tris-HCl pH 6.8 (Bio-Rad, Marnes-la-Coquette, France), 2% sodium dodecyl sulfate (SDS) (Sigma-Aldrich), 20% glycerol (Prolabo, Fontenay-sous-Bois, France), 1 mM sodium vanadate (Na_3_VO_4_, Labosi, Elancourt, France), 1 mM dithiothreitritol (DTT) (Merck, Fontenay Sous Bois, France) and 0.01% bromophenol blue (Sigma-Aldrich), sonicated and stored at -70°C. Proteins (50 μg) were separated on SDS-PAGE (10%) and transferred onto nitrocellulose membranes; membranes blocked with 5% dry skimmed milk in TBS were incubated with antibody overnight at 4°C. Anti-phosphotyrosine 705-STAT3 (1/1,000), anti-STAT3 (1/1,000), anti-NF-κB p50 (1/1,000), anti-NF-κB p65 (1/1,000), anti-STAT1 (1/1,000), and anti-OCT1 (1/1,000) were from Cell Signaling, anti-karyopherin/importin α (1:400) was from Santa Cruz (Tebu-bio, Le Perray en Yvelines, France). Blots were washed in TBS-T, incubated with peroxidase-coupled goat anti-mouse (Santa Cruz, Tebu-bio) or goat anti-rabbit (Upstate, Ozyme) secondary antibody (1/20,000) washed in TBS-T and revealed by chemiluminescence (LumiGLO reagent and peroxide; Cell Signaling) and autoradiography (X-Omat R film; Kodak). When necessary, membranes were stripped with Blot Restore Kit (Chemicon International) and reprobed with anti-actin antibody (Cell Signaling). Prestained molecular weight standards (Fermentas, Saint-Rémy-lès-Chevreuse, France) were used. For the quantification analysis, the bands from at least three separate experiments were scanned using a Chemidoc apparatus (Biorad) and quantification performed using the Quantity One software (Biorad). P-values were calculated using a t test.

### Real-time qPCR

The TaqMan^® ^Gene Expression Cells-to-CT™ kit (Applied Biosystems, Courtaboeuf, France) was used to extract total RNA and to perform reverse transcription and gene amplification. An Applied Biosystems Custom TaqMan Gene Expression Assay was used; the sequences were chosen to cover exons 5 and 6 to avoid detecting genomic DNA: sense primer: 5'-ccatcttcatcacactcttcctgtt, antisense primer: 5'-accaccgaggagaagatcca, 5'-FAM probe: 5-ctacagtgccaccgtcacc. For the TaqMan Gene Expression Assay (Applied Biosystems) ref. Hs00941525_g1 was used. For cyclophilin A (PPIA), used as a reference, the TaqMan Gene Expression Assay, ref. Hs99999904_m1 was used. All steps were performed following the recommendations of the manufacturer. Relative expression levels of each gene were calculated as previously described.

### Transfections

Cells were grown in 4-well plates to a density of 0.5 10^6 ^cells/mL. When the cells reached 50-60% confluence, they were transfected with STAT3-decoy ODN or the hairpin control decoy ODN (2 μg corresponding to 400 nM) in 150 μL of DMEM medium (without SVF) combined with the liposomes (2 μg of cationic lipid). After 6 h at 37°C in a humidified 5% CO_2 _incubator, the cells were placed in fresh serum-containing medium. Expression was analyzed after 48 h. In other cases, transfection was performed using polyethyleneimine (PEI, Sigma-Aldrich), with an ODN-to-polyethyleneimine ratio of 1:1.

### Flow cytometry, cell viability, immunocytochemistry

To measure cell death, cells were resuspended in annexin V-binding buffer, incubated with 5 μL of propidium iodide (BD Pharmingen, Morangis, France) and subjected to flow cytometry analysis, using a BD FACS Canto II Flow Cytometer. Cell viability was also assessed using the trypan-blue exclusion method with a V-cell counter (Beckmann, Villepinte, France).

For immunocytochemistry, cells were grown in 8-well plates (lab-tek, Nunc, Rochester, USA) to a density of 0.5 10^6 ^cells/mL. At 50-60% confluence, cells were transfected with the FITC-labeled STAT3-decoy ODN or the FITC-labeled mutated STAT3-decoy ODN. After 48 h the cells were washed in NaCl-phosphate buffer, fixed in 3.7% formaldehyde for 15 mn, permeabilized in 0.1% Triton X-100 for 15 mn and blocked in 5% FCS, 0.1% Tween in NaCl-phosphate buffer for 1 h. Cells were stained with anti-STAT3 antibody (Cell Signaling) (dilution: 1:100) or anti- phosphotyrosine 705-STAT3 antibody (Cell Signaling) (1:100) for 2 h and Alexa Fluor 546-labeled secondary antibody (Invitrogen) (1:200) for 90 mn. After counterstaining with 4', 6'- diamidino-2-phenylindole (DAPI) coverslips were mounted onto glass slides with Vectashield (Vectorlabs, Clinisciences, Montrouge, France). Fluorescence images were acquired using a Zeiss Axioplan2 Deconvolution microscope (Carl Zeiss, Le Pecq, France) and analyzed with Metafer4 (Metasystems, Altlussheim, Germany).

### Oligodeoxynucleotide pull-down

Nuclear protein extracts were obtained as follows: 20 million cells were resuspended in lysis buffer (20 mM Hepes, pH 7.4, 1 mM MgCl_2_, 10 mM KCl, 0.3% NP40, 0.5 mM dithiothreitol, 0.1 mM EDTA, protease inhibitors: Compete™, Boerhinger) at 4°C for 20 min. The lysates were centrifuged at 14 000 × g for 5 min at 4°C, and the supernatants containing the cytoplasmic proteins were discarded. The pellets were resuspended in the cell lysis buffer adjusted with 20% glycerol and 0.35 M NaCl for 30 min at 4°C. After centrifugation at 14 000 × g for 5 min at 4°C, the supernatants were stored at -80°C. For pull-down assays, 100-200 μg of nuclear protein extracts were incubated for 30 min at 4°C in binding buffer (1% NP40, 50 mM Hepes, pH 7.6, 140 mM NaCl) containing salmon sperm DNA (1 μg/assay) and 1 μg of the biotinylated hairpin decoy ODN or the mutated decoy ODN. The complexes were captured by incubation with 50 μl of avidin-Sepharose beads (neutravidin, Pierce) for 2 h at 4°C. For in-cell decoy ODN pull-down assays, the cells were first transfected with STAT3-decoy ODN or its mutated equivalent, as described under oligonucleotide transfection (see above), and then processed as above by cell lysis and recovery on avidin-Sepharose beads. After extensive washing with binding buffer, complexes were separated on SDS-polyacrylamide (8%) gel, and subjected to immunoblotting using an anti-STAT3 antibody (Cell Signaling). Results were analyzed by chemiluminescence (LumiGLO, Cell Signaling) and autoradiography (X-Omat R, Kodak).

### Antibody co-immunoprecipitation

For antibody pull-down assays, 20 million cells were lyzed and resuspended in lysis buffer (20 mM Hepes, pH 7.4, 1 mM MgCl_2_, 10 mM KCl, 1% NP40, 0.5 mM dithiothreitol, 0.1 mM EDTA, 1 mM orthovanadate, protease inhibitors; Compete™, Boerhinger) at 4°C for 5 min. The lysates were centrifuged at 14 000 × g for 5 min at 4°C, and the supernatants containing the cytoplasmic proteins were either used immediately or stored at -80°C. For the immunoprecipitations, 200 to 400 μg protein was supplemented with albumin-saturated protein G-agarose (Boehringer); after centrifugation (8000 × g, 5 min), the pellet was discarded and the supernatant conserved. Antibody was added (STAT3: 1:100; karyopherin, Santa-Cruz: 1:40) and incubation continued overnight at 4°C. Samples were then supplemented with albumin-saturated protein G-agarose and incubated for 1 h 30 m. The agarose beads were washed three times with TBS and once with TBS-T, and resuspended in SDS-sample buffer. Gel separation and western blotting were performed as described above.

## Results

### Survival of SW 480 colon carcinoma cells requires activated STAT3

**Figure 1:**
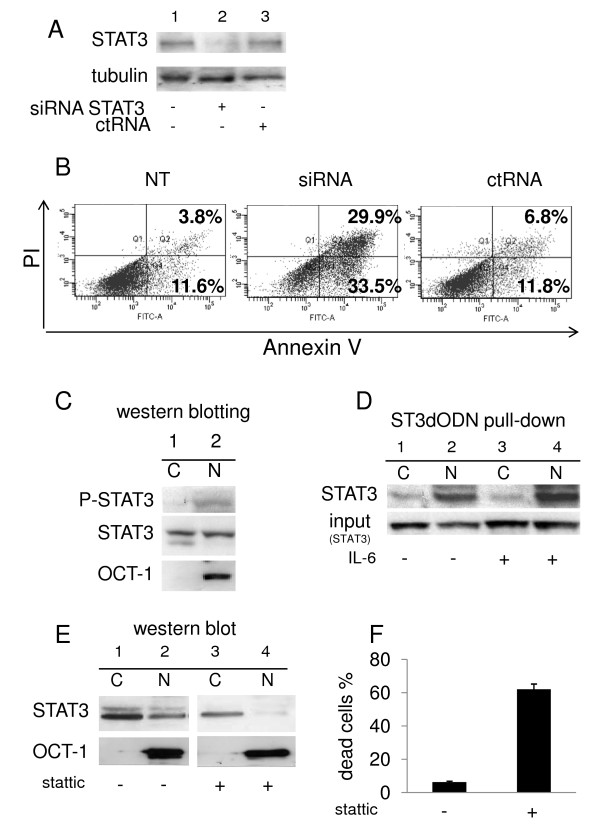
**STAT3 activation is required for SW 480 colon carcinoma cell survival**. **A**: Western blotting of STAT3 in cells not transfected with siRNA (lane 1) or transfected with either STAT3 siRNA (lane 2) or control siRNA (lane 3). **B**: Detection by cytometry of annexin V and propidium iodide (PI)-positive cells in the absence of transfection (NT), or after transfection with STAT3 siRNA (siRNA) or control siRNA (ctRNA). A typical experiment is shown. **C**: Western blotting of phospho-STAT3 and STAT3 in cytoplasmic (C) and nuclear (N) fractions of non-treated SW 480 cells. OCT-1 was used as nuclear marker control. **D**: Pull-down with biotinylated STAT3-decoy ODN using cytoplasmic (lanes 1 and 3) and nuclear (lanes 2 and 4) extracts from untreated (lanes 1 and 2) or IL-6-treated (50 ng/ml, 1 h) cells (lanes 3 and 4). **E**: Western blotting of phospho-STAT3 and STAT3 in cytoplasmic (C) and nuclear (N) fractions of non-treated SW 480 cells (1, 2) or Stattic-treated cells (20 μM). OCT-1 was used as nuclear marker control. **F**: Measurement of cell death using trypan blue-staining and counting with a V-cell automatic apparatus. Cells were either not treated or treated with Stattic (20 μM, 48 h).

STAT3 is activated in colon carcinoma and in the colon carcinoma cell line SW 480 [17,41]. In SW 480 cells transfected with specific STAT3 siRNA, the expression of STAT3 was strongly reduced (Figure 1A) and the number of annexin V-positive cells was significantly increased in comparison to cells treated with control siRNA (Figure 1B). STAT3 tyrosine 705 phosphorylation was detected (Figure 1C) as previously reported [17,41]. Pull-downs with biotinylated ODN were performed, followed by western blotting with anti-STAT3 antibody. This method is analogous to gel retardation assays, and revealed that STAT3 is activated. ODN-bound activated STAT3 was detected in nuclear extracts from untreated (Figure 1D, lane 2) and IL-6-treated cells (lane 4); cytoplasmic STAT3 only weakly bound the biotinylated ODN (Figure 1D, lanes 1 and 3). Treatment of cells with the STAT3 inhibitor Stattic (20 μM), known to inhibit STAT3 phosphorylation and dimerization [16], inhibited the nuclear translocation of STAT3 in SW 480 cells (Figure 1E, lane 4, see also figure threeD), as previously shown in other cell lines [16]; this correlated with induction of cell death (Figure 1F).

### Cytoplasmic sequestration of STAT3 and phospho-STAT3 by STAT3-decoy ODN

**Figure 2:**
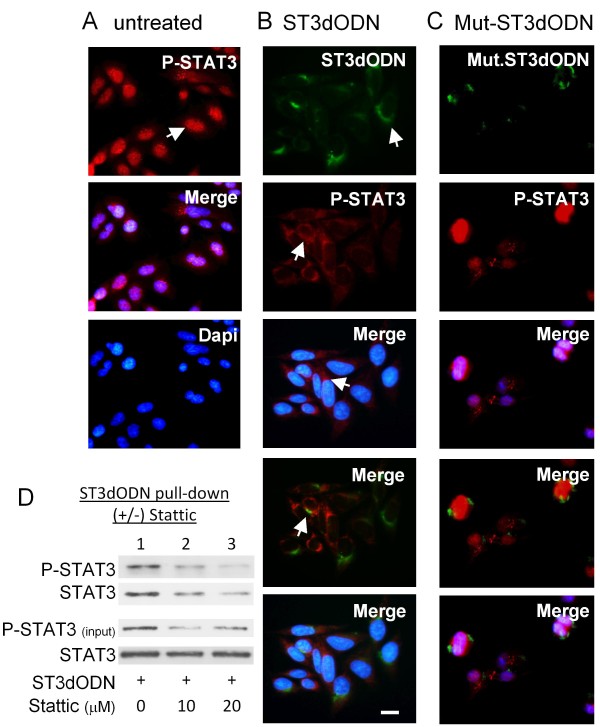
**Inhibition of phospho-STAT3 nuclear localization by STAT3-decoy ODN**. Immunofluorescence microscopy analysis: **A**: nuclear localization of phospho-STAT3 (red) in non-treated SW 480 cells. Cells were fixed and labelled with anti-phosphotyrosine 705-STAT3 antibody and counterstained with 4',6'-diamidino-2-phenylindole (Dapi); **B**: cytoplasmic localization of phospho-STAT3 (red) in STAT3-decoy ODN- transfected (2 μg/ml) SW480 cells; FITC-labeled STAT3-decoy ODN was detected in the cytoplasm (green), and cells counterstained with Dapi; **C**: nuclear localization of STAT3 (red) in mutated STAT3-ODN- transfected (2 μg/ml) SW480 cells; FITC-labeled mutated STAT3-ODN was detected in the cytoplasm (green), cells were counterstained with Dapi. Scale bar: 10 nm. In-cell STAT3-decoy ODN pull-down assays: **D**: Cells were transfected with biotinylated STAT3-decoy ODN, after cell lysis and recovery on avidin-Sepharose beads, complexes were subjected to western blotting using an anti-phospho-STAT3 antibody. Cells were either not treated (1) or treated with Stattic, 10 μM (2) or 20 μM (3).

**Figure 3:**
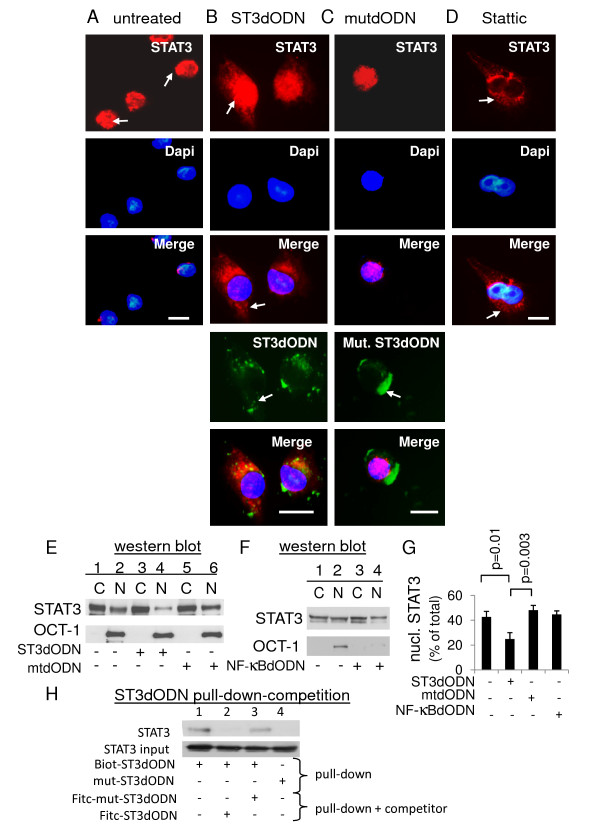
**Inhibition of STAT3 nuclear localization by STAT3-decoy ODN**. **A**: Nuclear localization of STAT3 (red) in non-treated SW 480 cells. Cells were fixed and labelled with anti-STAT3 antibody; **B**: cytoplasmic localization of STAT3 (red) in STAT3-decoy ODN- transfected (2 μg/ml) SW480 cells; FITC-labeled STAT3-decoy ODN was detected in the cytoplasm (green); **C**: nuclear localization of STAT3 (red) in mutated STAT3-ODN- transfected (2 μg/ml) SW480 cells; FITC-labeled mutated STAT3-ODN was detected in the cytoplasm (green); **D**: cytoplasmic localization of STAT3 in Stattic-treated cells (20 μM). Nuclei stained with DAPI. Scale: 10 nm. **E**: Cells transfected with STAT3-decoy ODN and STAT3 determined in cytoplasm and nucleus: no treatment (1 and 2), STAT3-decoy ODN 2 μg/ml (3 and 4), mutated STAT3-decoy ODN (mtODN) 2 μg/ml (5 and 6). OCT-1 antibody reprobing to check for cytoplasmic contamination. A typical result is shown. **F**: Cells were untreated (1 and 2), transfected with decoy NF-κB-ODN (2 μg/ml) (3 and 4), and STAT3 subcellular localization determined. **G**: Quantitative comparison. In control and mutated STAT3-decoy ODN (mtODN)- or decoy NF-κB-ODN-transfected cells, nuclear STAT3 (expressed as % of total STAT3, cytoplasmic + nuclear) was 50%; it was below 20% in STAT3-decoy ODN-transfected cells. Data from at least three independent experiments. A t test was used to calculate p values, n>3. **H**: Cells transfected with biotinylated STAT3-decoy ODN (1, 2 and 3) or its mutated equivalent (4) and complexes recovered and analyzed as in D; cells co-transfected with competitor: non-biotinylated STAT3-decoy ODN (2) or its mutated equivalent (3).

The subcellular distribution of STAT3-decoy ODN in SW 480 cells was shown by fluorescence microscopy to be essentially cytoplasmic (see [additional file 1], and figures 2B and 2C). Immunofluorescence microscopy analyzis of the subcellular localization of phospho-STAT3 in untreated SW480 cells showed that it was essentially nuclear (Figure 2A), but following STAT3-decoy ODN treatment it became mostly cytoplasmic (Figure 2B), this was not observed when using mutated STAT3-decoy ODN (Figure 2C). Pull-down experiments within cells transfected with biotinylated STAT3-decoy ODN followed by western blotting showed that phospho-STAT3 interacted with STAT3-decoy ODN (Figure 2D). In these experiments, the binding of phospho-STAT3 to the STAT3-decoy ODN was blocked by the treatment of cells with Stattic (10 and 20 μM), known to prevent STAT3 dimer formation [16], indicating that the STAT3-decoy ODN binds activated STAT3 dimers (Figure 2D). Total STAT3 was almost exclusively nuclear in untreated cells (Figure 3A), but after treatment with STAT3-decoy ODN it became cytoplasmic (Figure 3B). This was not observed when using mutated STAT3-decoy ODN (Figure 3C) but was observed when treating cells with Stattic (Figure 3D). Subcellular localization of STAT3 studied by cell fractionnation and western blotting showed that STAT3-decoy ODN efficiently prevented STAT3 nuclear translocation (Figure 3E, lane 4), whereas neither a mutated STAT3-decoy ODN (lane 6), nor a NF-κB-decoy ODN did so (Figure 3F, lane 4). A diagram of the data collected from several experiments illustrates the effect of STAT3-decoy ODN on the nucleo-cytoplasmic distribution of STAT3: in STAT3-decoy ODN-transfected cells nuclear STAT3 consisted of 20% of the total STAT3 (Figure 3G); conversely, in control-, mutated decoy ODN-, and NF-κB-decoy ODN-transfected cells, it consisted of 50% of the total STAT3, suggesting that STAT3-decoy ODN functions by trapping STAT3 within the cytoplasm. Finally, the binding of STAT3 to biotinylated STAT3-decoy ODN within cells was blocked by addition of excess non-biotinylated STAT3-decoy ODN (Figure 3H, and [additional file 2]).

### Cytoplasmic sequestration of STAT3 and phospho-STAT3 by STAT3-decoy ODN correlates with STAT3 inhibition and cell death

**Figure 4:**
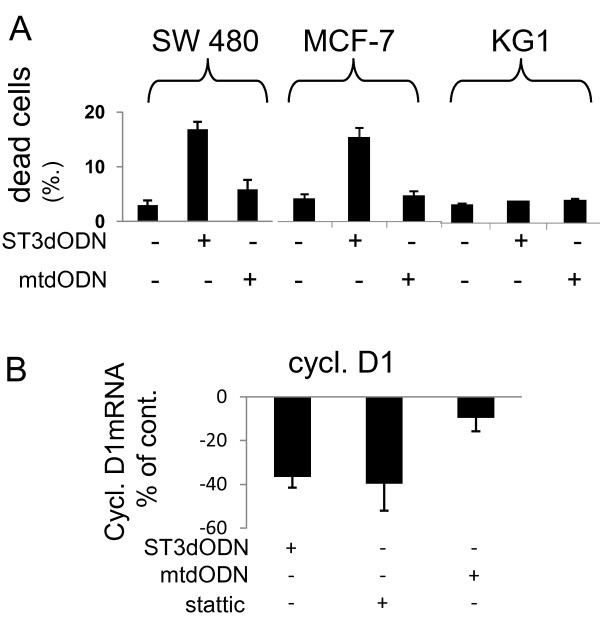
**Correlation of STAT3 nuclear translocation inhibition and cell death induction**. **A**: Cell death induction in STAT3-decoy ODN-transfected (2 μg/ml) SW 480 and MCF-7 cells, controls: no addition, mutated STAT3-decoy ODN (mtODN, 2 μg/ml); resistance of the acute myeloid leukemia cells KG1 to STAT3-decoy ODN (2 μg/ml), controls: non-transfected and mutated STAT3-decoy ODN (mtODN)-transfected (2 μg/ml) cells. Dead cells, incorporating trypan blue, were counted using the V-cell apparatus. Values are expressed in % of dead cells. **B**: Expression of cyclin-D1 in non-transfected, STAT3-decoy ODN-transfected (2 μg/ml), Stattic-treated (20 μM), and mutated STAT3-decoy ODN-transfected (2 μg/ml) SW 480 cells. Expression was measured using qPCR. Data, normalized using cyclophilin expression (ΔΔct), are expressed as % of control.

**Figure 5:**
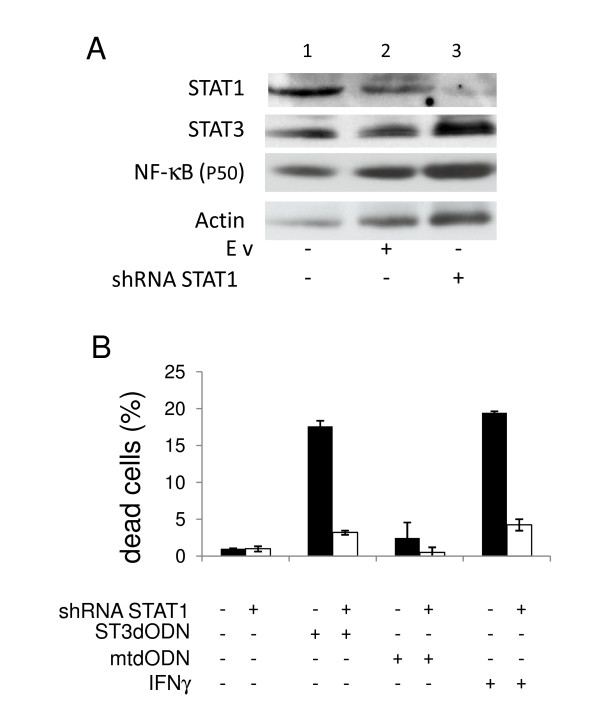
**Involvement of STAT1 expression level in STAT3-decoy ODN-induced cell death**. Colon carcinoma SW 480 cells transduced either with control virus (black histograms) or with STAT1-specific shRNA (white histograms). **A**: Detection of STAT1 by western blotting of control cells (1), cells transduced with an empty virus (Ev) (lane 2), and with a STAT1-specific shRNA lentivirus (3). Below are shown the detection of STAT3, the NF-κB (p50 subunit) and actin in the same extracts. **B**: Detection of dead cells by trypan blue exclusion counting. Control non-treated (black histograms) and STAT1-shRNA-treated cells (white histograms) were either not treated or transfected with STAT3-decoy ODN (ST3dODN) (48 h), mutated STAT3-decoy ODN (mtdODN) (48 h) or IFNγ (100 ng/ml, 48 h). Data are expressed in % of dead cells.

In STAT3-decoy ODN-transfected SW 480 cells, cell death increased (Figure 4A), as previously shown in these [17] and other cells [22-24]. STAT3-decoy ODN also induced cell death of MCF-7 cells, in which a low but detectable STAT3 activation has been previously observed [42]. However, the STAT3-decoy ODN had no effect on the acute myeloid leukemia cell line KG1, in which STAT5, rather than STAT3, is activated [43] (Figure 4A). The mutated STAT3-decoy ODN had no effect in any of the three cell lines (Figure 4A). The STAT3-target cyclin-D1 was analyzed by qPCR in STAT3-decoy ODN-transfected SW 480 cells: reduced cyclin-D1 expression was observed, similar to that observed with Stattic (Figure 4B). This suggests that it is the cytoplasmic trapping of STAT3 by the STAT3-decoy ODN which leads to cell death. Since STAT3-decoy ODN can also bind STAT1 and prevent STAT1-dependent IFNγ-induced cell death [17], experiments were performed to determine the overall involvement of STAT1 in STAT3-decoy ODN-induced cell death. To this end, STAT1 was silenced using shRNA in the SW 480 cells. As previously described in other cell systems [37], the expression of STAT1 was suppressed by specific shRNA, and not by empty vector; NF-κB and STAT3 expression was unchanged (Figure 5A). In STAT1-silenced SW 480 cells, the basal level of dead cells was unchanged. However, STAT3-decoy ODN-induced cell death was suppressed (Figure 5B). There was no effect of the mutated STAT3-decoy ODN, but IFNγ-induced cell death was suppressed (Figure 5B). Thus, STAT1 expression is important for cell death induction by STAT3 inhibitors, in agreement with observations published elsewhere [44], and in line with the notion that STAT1 is a key component of the cellular mechanism leading to cell death [45,46].

### Blockage of STAT3 nuclear transfer by STAT3-decoy ODN is overcome by IL-6-mediated activation of STAT3 or increased expression of recombinant STAT3

**Figure 6:**
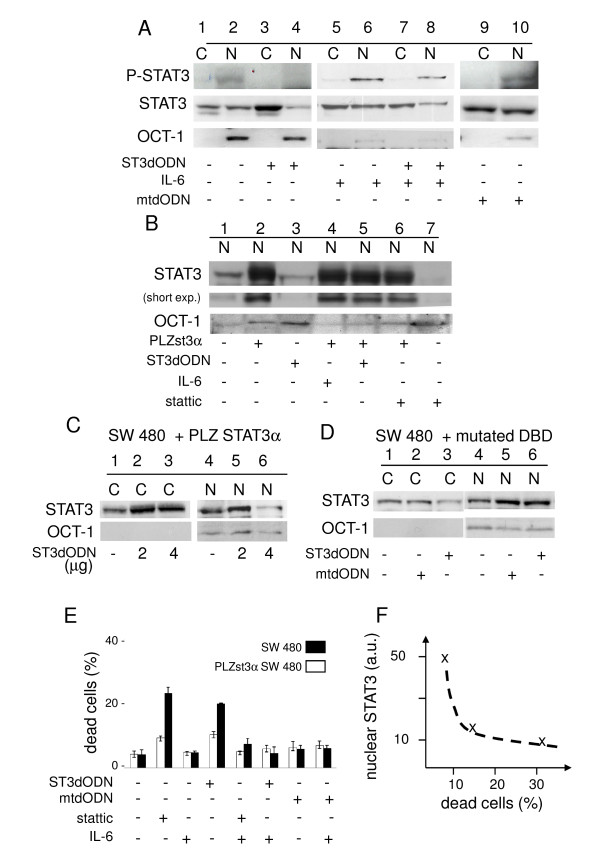
**Reversal of STAT3-decoy ODN-mediated inhibition of STAT3 nuclear translocation and cell death by IL-6 activation or overexpression of STAT3**. **A**: Nuclear STAT3 in IL-6- and STAT3-decoy ODN-treated cells. Non-treated (1, 2, 3 and 4), IL-6 treated (50 ng/ml, 16 h) (5, 6, 7 and 8) cells, non-transfected (1, 2, 5 and 6), STAT3-decoy ODN-transfected (2 μg/ml) (3, 4, 7 and 8) cells. **B**: nuclear STAT3 in STAT3-overexpressing cells transfected with STAT3-decoy ODN. Non-transfected (1, 3 and 7) and STAT3 plasmid (PLZst3α) (2, 4, 5 and 6) transfected cells. Stattic (6 and 7), STAT3-decoy ODN (lanes 3 and 5), or IL-6 (4 and 7) -treated cells. Diminished nuclear STAT3 in STAT3-overexpressing cells, treated with STAT3-decoy ODN or Stattic (5 μM) (compare 2, 5 and 6) (short exposure). (nuclear extracts are shown). **C**: Increased STAT3-decoy ODN prevents overexpressed STAT3 nuclear entry. ST3-PLZst3α plasmid-transfected cells transfected with 2 μg (2, 5), 4 μg (3, 6) or no (1, 4) decoy ODN. **D**: Nuclear accumulation of overexpressed DBD mutated STAT3: control (1 and 4), mutated STAT3-decoy ODN- (2 μg/ml) (2 and 5) or STAT3-decoy ODN- (2 μg/ml) (3 and 6) transfected cells. **E**: Cell death (trypan blue exclusion) in control (black histograms) and STAT3-overexpressing cells (white histograms). Non-treated or Stattic- (5 μM), STAT3-decoy ODN- (2 μg/ml, 48 h) (ST3dODN), Stattic (5 μM) and IL-6- (50 ng/ml), mutated STAT3-decoy ODN- (2 μg/ml, 48 h) (mtdODN), or mutated STAT3-decoy ODN (2 μg/ml, 48 h) (mtdODN) and IL-6- (50 ng/ml, 48 h) treated cells (data in % of dead cells). **F**: Summary plot for A, B and C showing inverse nuclear STAT3/dead cells correlation (a.u.: arbitrary units).

To evaluate the relevance of STAT3-decoy ODN/STAT3 interaction in the reduction of nuclear STAT3, cells were transfected with STAT3-decoy ODN and treated with IL-6, and nuclear STAT3 was analyzed. Treatment with IL-6 significantly increased the tyrosine phosphorylation of STAT3; inhibition of STAT3 nuclear transfer by STAT3-decoy ODN was partially overcome by this treatment (Figure 6A), suggesting a titrating effect of the increased amount of active STAT3 on STAT3-decoy ODN action. Similarly, overexpression of STAT3 by plasmid transfection of SW 480 cells resulted in a marked increase of STAT3 nuclear localization (Figure 6B, lane 2) and considerably reduced inhibitory effects of STAT3-decoy ODN (lane 5) and Stattic (lane 6). However, when more STAT3-decoy ODN (4 μg) was added to STAT3-overexpressing cells, a reduction of nuclear STAT3 was observed (Figure 6C, lane 6). Conversely, in cells transfected with DBD-mutated STAT3, there was no change in nucleo-cytoplasmic distribution after treatment with STAT3-decoy ODN (Figure 6D, lane 6). This emphasizes the notion that a functional DBD is necessary for STAT3-decoy ODN to prevent STAT3 nuclear localization. Cell death was also analyzed to determine whether modifications of STAT3's subcellular localization affected cell fate. Overexpression of STAT3 in SW 480 cells significantly reduced the rate of cell death induced by STAT3-decoy ODN or by Stattic (5 μM) (Figure 6E). Combining the cell death data shown in Figure 6E with the amounts of nuclear STAT3 of Figure 6B showed that nuclear accumulation of STAT3 is inversely proportional to cell death (Figure 6F). Overall, these experiments suggest that STAT3-decoy ODN-induced cell death results from the inhibition of STAT3 through its entrapment within the cytoplasm.

### STAT3-decoy ODN interferes with the cytoplasmic-nuclear shuttling of STAT3

**Figure 7:**
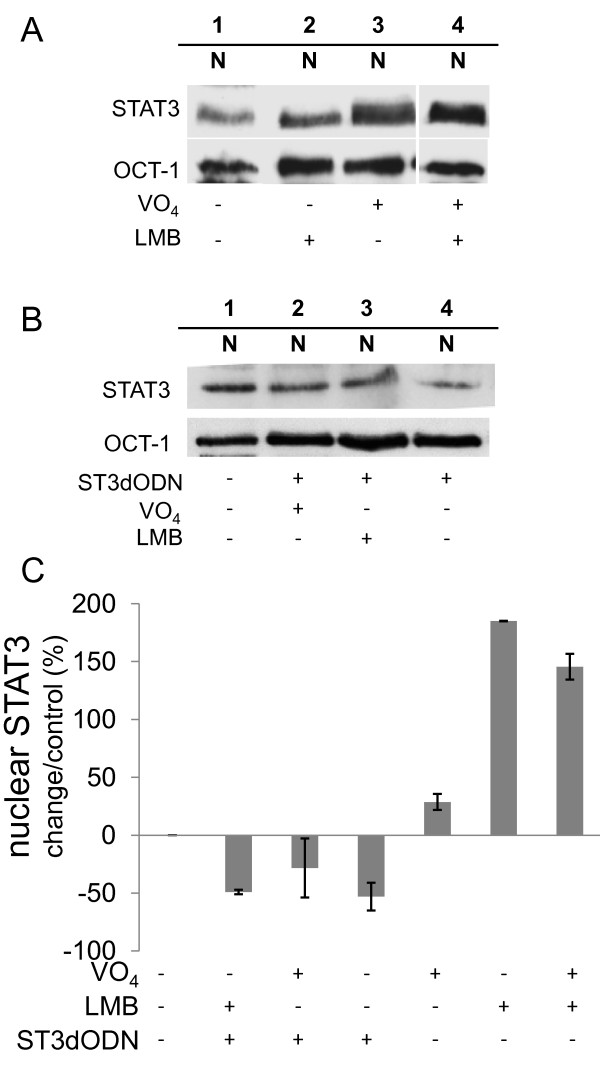
**The CRM1 inhibitor leptomycin B and the phosphatase inhibitor vanadate reverse inhibition of STAT3 nuclear translocation by STAT3-decoy ODN**. **A**: Amounts of nuclear STAT3 following treatment of cells with leptomycin (LMB) or vanadate. Cells were either not treated (1) or treated with LMB (10 ng/ml, 3 h) (2), sodium vanadate (100 μM, 2 h) (3), or both (4); for clarity, only nuclear extracts (N) are shown. **B**: Effect on STAT3 nuclear localization of cells' simultaneous treatment with STAT3-decoy ODN, LMB, and vanadate. Cells were either not transfected (1) or transfected with STAT3-decoy ODN alone (2 μg/ml) (4), STAT3-decoy ODN (2 μg/ml) and vanadate (100 μM) (2), or STAT3-decoy ODN (2 μg/ml) and LMB (10 ng/ml, 3 h) (3). Only nuclear extracts are shown (N). **C**: Quantitative analysis of nuclear STAT3 from several experiments identical to those shown in A and B. STAT3 amounts quantified by scanning are expressed relative to control (in %). Conditions were: STAT3-decoy ODN (ST3dODN) and vanadate (VO_4_), STAT3-decoy ODN and LMB, STAT3-decoy ODN alone, vanadate, LMB, vanadate and LMB. Data are from at least three separate experiments.

Nuclear transfer of activated STAT3 through nuclear pores is dependent on nuclear import involving karyopherin/importins, which interact with STAT3's NLS; the NLS located within the DBD being the most efficient in STAT3 [3,5]. The activated STAT3 dimer enters the nucleus, binds its DNA targets, and is then released, after which it is dephosphorylated by a nuclear tyrosine phosphatase [7]; inhibition of the phosphatase with vanadate traps STAT3 in the nucleus (shown by increased nuclear phospho-STAT3 in vanadate-treated SW480 cells, see supplemental data 3). The transfer of STAT3 to the cytoplasm depends in part on the export protein CRM1 [47], which can be selectively inhibited by leptomycine B (LMB) [48]. To further characterize the mechanism of action of STAT3-decoy ODN on STAT3 nucleo-cytoplasmic shuttling, the cells were treated with LMB or vanadate. Treatment with LMB (10 ng/ml) increased nuclear STAT3 (Figure 7A, lane 2, and 7C), as previously shown in v-src expressing cells [31] (it also inhibited STAT3 phosphorylation, in agreement with previous results [31], see [additional file 3]). Treatment with vanadate (100 μM) or with both LMB and vanadate also increased nuclear STAT3 (Figure 7A, and 7C). Vanadate (Figure 7B, lane 2) and LMB (Figure 7B, lane 3) competed with STAT3-decoy ODN and opposed its action by retaining STAT3 within the nucleus.

**Figure 8:**
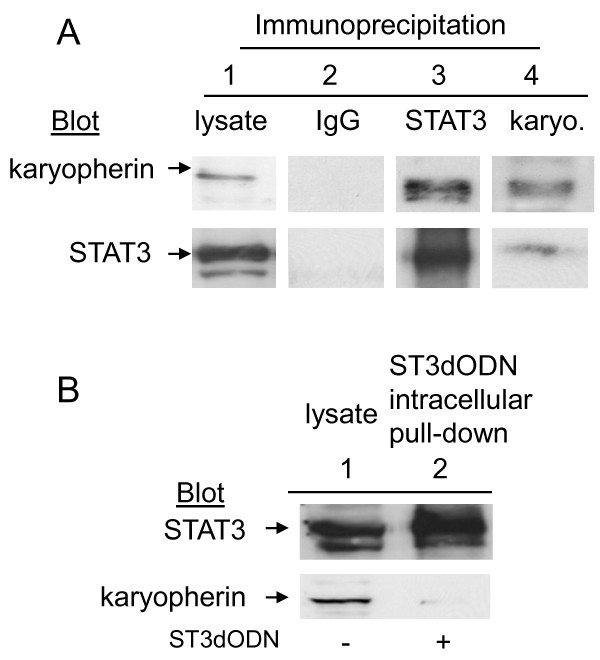
**Interaction of STAT3 with the importin/karyopherin complex is impaired by STAT3-decoy ODN**. **A**: Co-immunoprecipitation of STAT3 with karyopherin α. Cells were lysed and immunoprecipitation performed with anti-STAT3 (1:100) (3) or with anti-karyopherin α (1:40) (4) antibodies. A control experiment was performed with non-relevant immunoglobulin (IgG, 1:50) (2). After separation on gel and transfer, the membrane was probed with anti-karyopherin α (1:400) or anti-STAT3 antibody (1:1000). A control cell lysate was loaded on the gels (1). **B**: Intra-cellular pull-down assays with the biotinylated STAT3-decoy ODN. Cells were transfected with biotinylated STAT3-decoy ODN (6 h) and complexes collected with avidin agarose and analyzed by western blotting. Control SW 480 cell lysate (1), STAT3-decoy ODN (ST3dODN) pull-down (2). Membranes were probed with antibody to STAT3 and reprobed with antibody to karyopherin α.

Nuclear transfer of activated STAT3 involves karyopherins/importins which interact with the NLS located within the DBD [3,5]. To verify the possibility of a competition between karyopherin and STAT3-decoy ODN for STAT3 binding, STAT3 immunoprecipitation and STAT3-decoy ODN pull-downs were compared to one another for their karyopherin content. Karyopherin was detected in the STAT3 immunoprecipitates (Figure 8A, lane 3) and STAT3 was detected in the karyopherin immunoprecipitates (lane 4), but neither of them was detected in the IgG immunoprecipitate control (lane 2). On the other hand, STAT3-decoy ODN-trapped STAT3, detected by treating cells with biotinylated STAT3-decoy ODN (under the conditions shown above to inhibit STAT3 nuclear transfer; see Figure 2A), collected on avidin-coated agarose beads and analyzed by western blotting, contained either no karyopherin or only trace amounts, while clearly containing STAT3 (Figure 8B, lane 2), as previously shown [17]. An identical experiment performed with mutated STAT3-decoy ODN showed practically no detectable STAT3 or karyopherin (not shown). The amount of karyopherin detected in the crude lysates is shown in figures 8A (lane 1), and 8B (lane 1). The observations reported here suggest that STAT3-decoy ODN impairs the binding of STAT3 complexes to karyopherin.

### Inhibition of STAT3 with STAT3-decoy ODN in SW 480 cells is associated with inhibition of NF-κB

**Figure 9:**
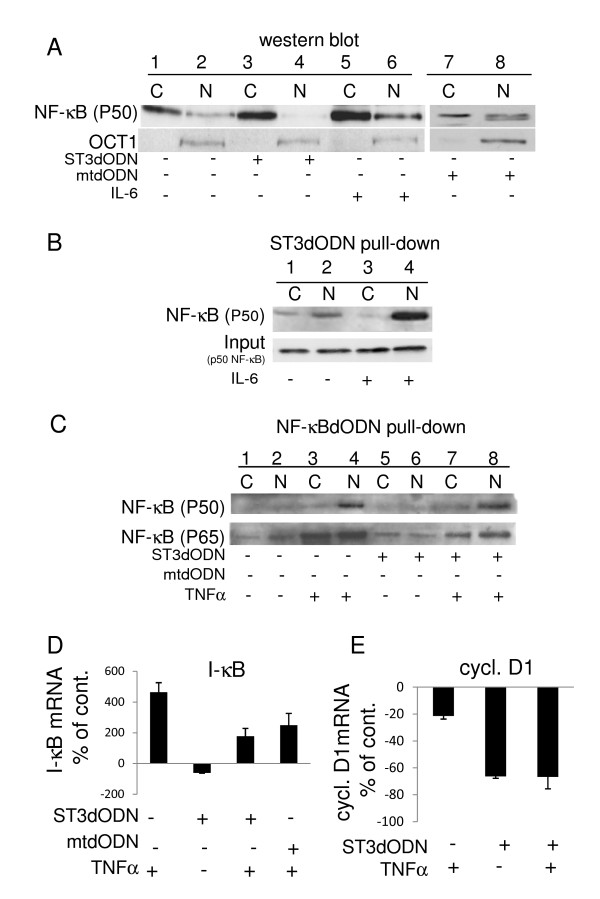
**Functional interaction of STAT3 with NF-κB in SW 480 cells**. **A**: NF-κB nuclear transfer in STAT3-decoy ODN-transfected SW 480 cells. Cells were transfected with STAT3-decoy ODN (ST3dODN) (2 μg/ml) (3 and 4), IL-6 (50 ng/ml, 16 h) (5 and 6), or mutated STAT3-decoy ODN (mtODN) (7 and 8). Controls: 1 and 2. **B**: Probe of STAT3-decoy ODN pull-down with NF-κB antibody in non-transfected cytoplasm and nuclear fractions (1 and 2) and in IL-6 (50 ng/ml)-treated cytoplasm and nuclear fractions (3 and 4). **C**: Analysis of NF-κB activity by pull-down assay with NF-κB-decoy ODN following treatment of cells with STAT3-decoy ODN (2 μg/ml). Cells treated with TNF-α (20 ng/ml) (3 and 4) transfected with STAT3-decoy ODN (2 μg/ml) (lanes 5 and 6), treated with both TNF-α and STAT3-decoy ODN (7 and 8), transfected with mutated STAT3-decoy ODN (2 μg/ml) (9 and 10), or with both TNF-α and mutated STAT3-decoy ODN (11 and 12); untreated cells: 1 and 2. Western blotting with NF-κB antibody (p50 or p65 subunits). **D**: mRNA levels of the NF-κB target I-κB (qPCR) after STAT3-decoy ODN treatment. Cells were not transfected, transfected with STAT3-decoy ODN (ST3dODN) (2 μg/ml), treated with TNF-α (20 ng/ml), treated with both STAT3-decoy ODN and TNF-α, or transfected with mutated STAT3-decoy ODN (mtdODN) (2 μg/ml) and TNF-α. **E**: mRNA levels of cyclin-D1 (qPCR) after treatment with STAT3-decoy ODN. Cells were not treated, treated with TNF-α (20 ng/ml), transfected with STAT3-decoy ODN (2 μg/ml), or treated with both TNF-α and STAT3-decoy ODN. Results are expressed as % of control.

Experiments were conducted to determine whether the inhibition of STAT3 by STAT3-decoy ODN could indirectly affect NF-κB nuclear translocation, since a functional interaction between STAT3 and NF-κB has been reported in several cellular systems [21,34,49]. In the SW 480 cells, Stattic was found to inhibit the nuclear transfer of both STAT3 and NF-κB (not shown), and to inhibit NF-κB activity as measured with a NF-κB-specific reporter plasmid (not shown). STAT3-decoy ODN, but not mutated decoy ODN, markedly reduced NF-κB nuclear translocation (Figure 9A, lanes 4 and 8). Pull-down experiments with STAT3-decoy ODN also showed higher amounts of NF-κB in the complex obtained from the nuclear fraction of IL-6-treated cells (Figure 9B, lane 4, and [additional file 4]). TNF-α-stimulated nuclear transfer of NF-κB (Figure 9C, lane 4), which was inhibited by the NF-κB-decoy ODN (not shown), was insensitive to STAT3-decoy ODN (Figure 9C, lane 8); similar results were observed when probing the NF-κB pull-downs with anti-p65 antibody, except that the basal levels were somewhat higher (Figure 9C). The expression of the direct NF-κB target I-κB, as determined by qPCR analysis, was inhibited to 50% of the control level by STAT3-decoy ODN (Figure 9D). However, TNF-α-induced I-κB mRNA remained high in the presence of STAT3-decoy ODN (Figure 9D). On the other hand, the expression of cyclin D1, a STAT3 target gene, was inhibited by STAT3-decoy ODN (Figure 9E). Thus, a decoy ODN targeting STAT3 can inhibit NF-κB indirectly.

## Discussion

**Figure 10:**
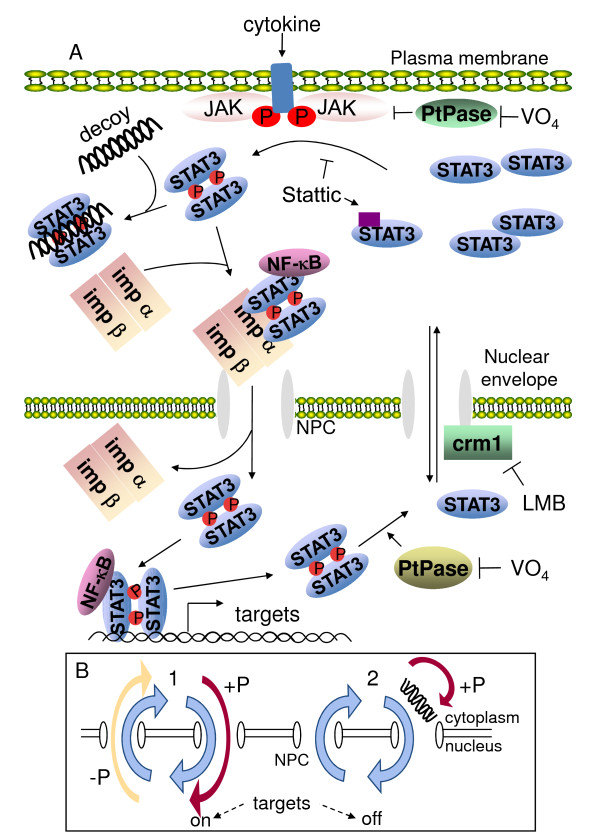
**Nucleo-cytoplasmic shuttling of STAT3, and putative mechanism of action of STAT3-decoy ODN**. **A**: Nucleo-cytoplasmic shuttling of activated STAT3. Phosphorylation of STAT3 on tyrosine 705 by the JAK family kinases results in dimerization and interaction with importins (imp), followed by transfer to the nucleus through the nuclear pore complex (NPC). Phospho-STAT3 binds its DNA targets, this is followed by dephosphorylation by a nuclear tyrosine phosphatase. Unphosphorylated STAT3 re-enters the cytoplasm: this depends in part on CRM1, which is inhibited by leptomycin B (LMB). Tyrosine phosphatases which dephosphorylate activated JAKs and phospho-STAT3 are inhibited by sodium vanadate (VO_4_). Stattic interaction with STAT3 monomers prevents dimerization and nuclear entry. STAT3-decoy ODN (decoy) interaction with active phosphorylated STAT3 dimers is suggested to compete with importin, thereby trapping active STAT3 in the cytoplasm. **B**: Nucleo-cytoplasmic cycling of activated and non-activated STAT3. Non-activated STAT3 cycles in and out of the nucleus in the absence of activation (phosphorylation: +P) (1, blue arrows). Activated STAT3 enters the nucleus by a transporter-mediated process (1, red arrow) and returns to the cytoplasm following dephosphorylation: -P) (1, yellow arrow). In STAT3-decoy ODN-transfected cells, cycling of non-activated STAT3 is unchanged (2, blue arrows), whereas activated (phosphorylated) STAT3 does not enter the nucleus (2, red arrow). NPC: nuclear pore complex. The scheme in B is adapted from a figure of ref.[3].

Constitutive STAT3 activation is frequently involved in uncontrolled tumor cell proliferation and therefore constitutes a valuable target for anti-tumor therapy [11,50]. Decoy oligonucleotides (decoy ODNs) have been shown to efficiently induce cell death in many different cellular systems (see [51] for review) and to have a potential for specific targeting of tumor cells. However, although it is generally assumed that decoy ODNs must enter the nucleus to exert their inhibitory action on the targeted TF, little is known of their mechanism of action. To study this issue, the SW 480 colon carcinoma tumor cell line was used as a model system. These cells require a basal level of activated STAT3 for survival [17,41], as confirmed here by STAT3-specific siRNA- or Stattic-induced cell death. To analyze the impact of a STAT3-decoy ODN on the subcellular localization of STAT3 in the colon carcinoma cell line SW 480, a combination of subcellular fractionation, oligonucleotide pull-down and immunofluorescence microscopy was used, in non-stimulated cells in most experiments in order to reproduce physiological conditions as much as possible. The main observations of this study are that: i, the STAT3-decoy ODN interacts with active phospho-STAT3 dimers, ii, this interaction results in the cytoplasmic trapping of phospho-STAT3 and iii, blocked nuclear transfer of active STAT3 by STAT3-decoy ODN results in reduced cyclin D1 (a key target of STAT3) expression and cell death induction. STAT3-decoy ODN was found to interact with activated dimeric STAT3 but not with non-activated STAT3 (see Figure 1A where STAT3, although present in the cytoplasmic fraction, does not interact with the STAT3-decoy ODN and the pull-down assays of Figure 2 where phospho-STAT3 is brought down by the STAT3-decoy ODN but not in the presence of Stattic). The specificity of STAT3 targeting by STAT3-decoy ODN was further evidenced by the absence of any effect on STAT3 nuclear transfer of either a control mutated STAT3-decoy ODN or a decoy NF-κB-ODN. In addition, STAT3-decoy ODN did not prevent the nuclear transfer of DNA binding domain (DBD)-mutated STAT3, indicating that a functional DBD domain is necessary for activated STAT3 to bind STAT3-decoy ODN. Nuclear entry of proteins involves a NLS within the protein's sequence, which allows interaction with components of the nuclear membrane pore complex (NPC). About half of the identified NLS lie within the DBD of proteins. STAT3 contains several NLS [32,52], but its major functional NLS lies within the DBD [3,5]; this dimer-specific NLS appears to be essential for STAT dimer binding to karyopherin/importins [53] which mediate interaction with the NPC. This suggests that STAT3 cytoplasmic trapping by STAT3-decoy ODN results from the interaction of the decoy ODN with the DBD domain of a functional STAT3 dimer, thereby masking the NLS and preventing interaction with karyopherin/importins as depicted in Figure 10A. Indeed, STAT3 immunoprecipitation brought down karyopherin α, while STAT3-decoy ODN pull-down did not. This observation is in agreement with studies showing that an ODN containing the m67 sequence can displace the interaction of recombinant phospho-STAT1 with recombinant importin α [54]. The observation that STAT3-decoy ODN inhibited only activated STAT3 suggests that it could effectively inhibit STAT3 in cells in which there is basal nucleo-cytoplasmic shuttling of activated STAT3. Constitutive nucleo-cytoplasmic shuttling of STAT3 has been observed in several cell systems [52], including v-src transformed cells in which STAT3 is activated [31]. Previous immuno-cytochemistry studies have shown that the CRM-1 inhibitor LMB interferes with the nucleo-cytoplasmic shuttling of STAT3, resulting in its nuclear accumulation [8,31] and inhibition of tyrosine phosphorylation [31]. In the present study, LMB increased nuclear localization of STAT3 and prevented the action of STAT3-decoy ODN, reinforcing the view that the latter acts on nucleo-cytoplasmic shuttling. Similar results were obtained with the tyrosine phosphatase inhibitor vanadate, which increased the amount of nuclear STAT3, and also prevented the action of STAT3-decoy ODN, thereby preventing cytoplasmic retention of STAT3. The modification of total STAT3 subcellular distribution induced by STAT3-decoy ODN observed here suggests that STAT3-decoy ODN functions by targeting the nucleo-cytoplasmic traffic itself, as previously suggested [33]. The data also indicate that STAT3-decoy ODN impairs the shuttling of active STAT3 dimers only, without interfering with the previously reported constitutive activation-independent nucleo-cytoplasmic shuttling of non-phosphorylated dimers [32,55,56] (see Figure 10). Non-phosphorylated STAT3 was found to be transcriptionally active [57], but whether it can bind STAT3 consensus sequences is not clear [58]. Thus, in all likelihood, the inhibitory action of STAT3-decoy ODN relies on the constitutive activation of STAT3 in SW 480 cells leading to its nuclear localization. The specific inhibition of the nucleo-cytoplasmic turn-over of STAT3 distinguishes STAT3-decoy ODN from chemicals, such as Stattic [16], that have been designed to prevent STAT3 dimer formation by interacting with the SH2 domain, subsequently impairing nuclear translocation; and from siRNAs, which target the entire STAT3 protein pool in any cell, disrupting functions that may be unrelated to growth and increasing the chances of side effects.

Cell death and apoptosis depend partly on STAT1-dependent effector genes [46,59] (see also [60] for a review). In the present study, the suppression of STAT1 by RNA silencing prevented cell death induction by STAT3-decoy ODN, indicating a critical role for STAT1, independent of IFNγ activation, in line with previous observations showing STAT1-dependent IFN-independent cell death [59]; (see also: [61]). However, STAT1 is the major effector of IFNγ, which is antiproliferative or tumoricidal in several cancer cell types [62,63]; although STAT3-decoy ODN has been found to induce tumor cell death in several different cell systems [17,22-24], it can also inhibit STAT1 [17,64]. Despite their opposing biological effects [65], STAT1 and STAT3 form heterodimers whose function is unclear [66]. Thus, despite its efficiency in inducing cell death, and although it has been found to have few side effects when administered to primates [67], suggesting a potential for clinical applications, STAT3-decoy ODN must be optimized so that it can distinguish between STAT3 and STAT1. Work is in progress to try and define the structural constraints that underlie specific recognition of STAT3-decoy ODN by STAT3.

In cancer, STAT3 and NF-κB have been shown to cooperate in promoting cell growth by interacting at different levels of their activating pathways [35]. STAT3 can trap constitutively activated NF-κB within the nucleus of tumor cells [68]. In SW 480 cells, both NF-κB and STAT3 are activated, suggesting a constitutive interleukin secretory loop, as described for several tumor cell systems (see: [49]). The findings of the present paper indicate that active STAT3 interacts with NF-κB in the colon-carcinoma cell line SW 480, as shown by the presence of NF-κB in STAT3-decoy ODN pull-downs and by reduced NF-κB transcriptional activity. Unphosphorylated STAT3 also interacts with NF-κB, but apparently binds κB sites [57], and may not be recognized by STAT3-decoy ODN for this reason. Thus, by trapping active STAT3 within the cytoplasm, STAT3-decoy ODN can simultaneously trap the fraction of NF-κB that is associated to active STAT3; this may potentially allow the targeting of a subset of genes that is essential for uncontrolled tumor cell growth.

## Conclusions

STAT3-decoy ODN is an efficient inducer of cell death in the colon-carcinoma cell line SW 480. It is shown here to function by trapping activated STAT3 within the cytoplasm by binding to the active dimer and preventing binding to karyopherin, which is required for the transfer of active STAT3 into the nucleus. STAT3-decoy ODN appears to be capable of specifically targeting active STAT3, and not its inactive form. Thus, STAT3-decoy ODN inhibits STAT3 only in cells where STAT3 is activated, such as cancer cells, resulting in cell death without harming healthy cells. Furthermore, the entrapment of STAT3-bound NF-κB adds a new powerful anticancer potential to STAT3-decoy ODN. These results point to the DNA binding domain of STAT3, as well as the process through which activated STAT3 enters the nucleus, as potential sources of active anti-cancer compounds.

## Authors' contributions

IS carried out the biochemical, molecular, and cell biology studies. VM, FBM, CR, IN, POS, DL, LAK and SLC carried out part of the experiments. IDF, AC, and NVB contributed essential reagents. IS and RF designed the study and wrote the paper. All authors read and approved the final manuscript.

## Supplementary Material

Additional file 1 **Subcellular localization of the STAT3-decoy ODN**. Cells were grown in 8-well plates to a density of 2.104 cells/mL. When the cells reached 50-60% confluence, they were transfected with the FITC-labeled (green) STAT3-decoy ODN (2 μg) in 150 μL of culture medium (DMEM without Fetal Calf Serum) combined to the liposomes (2 μg of cationic lipid). After 6 h at 37°C in a humidified 5% CO2 incubator, the cells were placed in fresh FCS-containing medium. After 48 h the cells were fixed and stained with DAPI to visualize nuclei and examined by fluorescence microscopy (A: nuclei, B: merge, C: FITC-labeled decoy). Click here for file

Additional file 2 **In-cell STAT3-decoy ODN pull-down assays**. Cells were transfected with the STAT3-decoy ODN, as described under oligonucleotide transfection (see methods), and then processed by cell lysis and recovery on avidin-Sepharose beads. After extensive washing with binding buffer, complexes were separated on SDS-polyacrylamide (8%) gel, subjected to immunoblotting using an anti-phospho-STAT3 antibody (Cell Signaling); input was determined by analyzing an aliquot of the initial lysate with STAT3 antibody (Cell Signaling). Results were analyzed by chemiluminescence (LumiGLO, Cell Signaling) and autoradiography (X-Omat R, Kodak). In A, cells were either not treated (1) or treated with decoy STAT3-ODN (2). In B, cells were either not treated or treated with IL-6 (50 ng/ml). Click here for file

Additional file 3 **Effect of leptomycin B and of vanadate on the level of phospho-STAT3**. Cells were either not treated (1, 2), treated with leptomycin B (LMB) (5 ng/ml) (3, 4), (10 ng/ml) (5, 6), (15 ng/ml) (7, 8) or vanadate (200 μM) (9, 10) (500 μM) (11, 12), for 4 h. Cytoplasmic (C) and nuclear extracts (N) (see methods) were analyzed on acrylamide gels and the membranes probed with anti-phospho-STAT3 and anti-Oct-1 antibodies. Click here for file

Additional file 4 **Effect of the STAT3-decoy ODN and of IL-6 on the nuclear localization of the p50 subunit of NF-κB**. Cells were either not treated (1, 2), treated with STAT3-decoy ODN (2 μg/ml) (3, 4), IL-6 (50 ng/ml) (5, 6) or both (7, 8) for 6 h. Cytoplasmic (C) and nuclear extracts (N) (see methods) were analyzed on acrylamide gels and the membranes probed with anti-p50-NF-κB and anti-Oct-1 antibodies. Click here for file
