# Supplementary figures and images for: A STAT3-decoy oligonucleotide induces cell death in a human colorectal carcinoma cell line by blocking nuclear transfer of STAT3 and STAT3-bound NF-κB
Source: BMC Cell Biol. 2011 Apr 12;12:14. doi: 10.1186/1471-2121-12-14 (PMC3082224; doi:10.1186/1471-2121-12-14)

## Slide 1
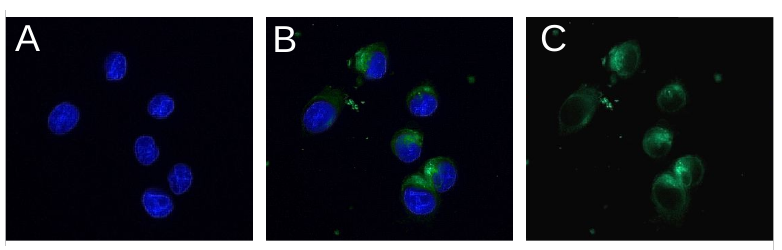

A
C
B

Supplement: Additional file 1 — Subcellular localization of the STAT3-decoy ODN. Cells were grown in 8-well plates to a density of 2.104 cells/mL. When the cells reached 50-60% confluence, they were transfected with the FITC-labeled (green) STAT3-decoy ODN (2 μg) in 150 μL of culture medium (DMEM without Fetal Calf Serum) combined to the liposomes (2 μg of cationic lipid). After 6 h at 37°C in a humidified 5% CO2 incubator, the cells were placed in fresh FCS-containing medium. After 48 h the cells were fixed and stained with DAPI to visualize nuclei and examined by fluorescence microscopy (A: nuclei, B: merge, C: FITC-labeled decoy). [file 1471-2121-12-14-S1.PPT]

## Slide 1
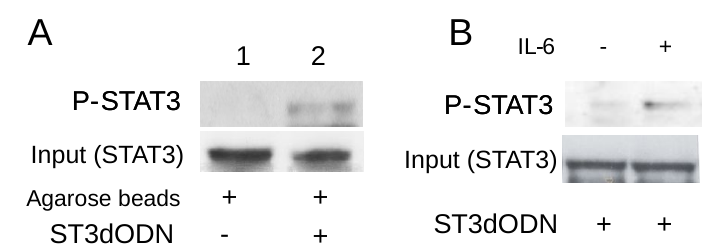

A
B
IL
-
-
6
-
+
1 2
P
P
-
-
STAT3
STAT3
P
P
-
-
STAT3
STAT3
Input (STAT3)
Input (STAT3)
+
+
Agarose beads
ST3dODN
+ +
ST3dODN
-
+

Supplement: Additional file 2 — In-cell STAT3-decoy ODN pull-down assays. Cells were transfected with the STAT3-decoy ODN, as described under oligonucleotide transfection (see methods), and then processed by cell lysis and recovery on avidin-Sepharose beads. After extensive washing with binding buffer, complexes were separated on SDS-polyacrylamide (8%) gel, subjected to immunoblotting using an anti-phospho-STAT3 antibody (Cell Signaling); input was determined by analyzing an aliquot of the initial lysate with STAT3 antibody (Cell Signaling). Results were analyzed by chemiluminescence (LumiGLO, Cell Signaling) and autoradiography (X-Omat R, Kodak). In A, cells were either not treated (1) or treated with decoy STAT3-ODN (2). In B, cells were either not treated or treated with IL-6 (50 ng/ml). [file 1471-2121-12-14-S2.PPT]

## Slide 1
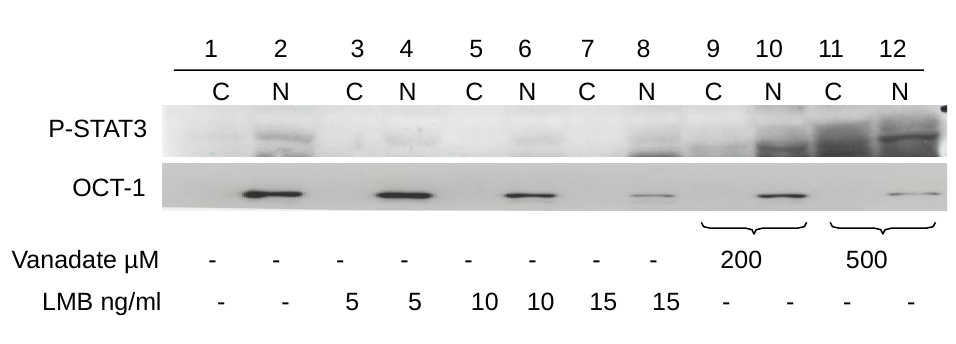

1 2 3 4 5 6 7 8 9 10 11 12
 C N C N C N C N C N C N
P-STAT3
OCT-1
Vanadate µM - - - - - - - - 200 500
 LMB ng/ml - - 5 5 10 10 15 15 - - - -

Supplement: Additional file 3 — Effect of leptomycin B and of vanadate on the level of phospho-STAT3. Cells were either not treated (1, 2), treated with leptomycin B (LMB) (5 ng/ml) (3, 4), (10 ng/ml) (5, 6), (15 ng/ml) (7, 8) or vanadate (200 μM) (9, 10) (500 μM) (11, 12), for 4 h. Cytoplasmic (C) and nuclear extracts (N) (see methods) were analyzed on acrylamide gels and the membranes probed with anti-phospho-STAT3 and anti-Oct-1 antibodies. [file 1471-2121-12-14-S3.PPT]

## Slide 1
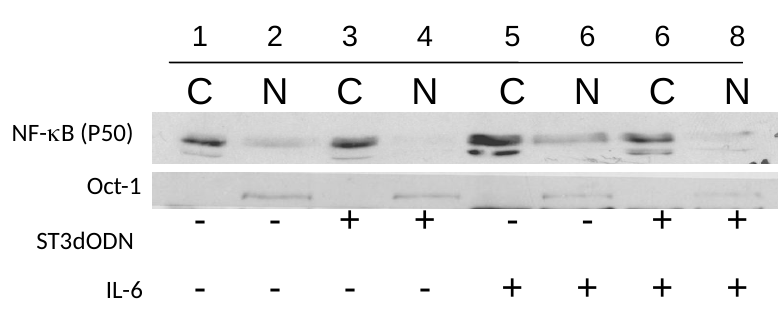

1
C
-
-
2
N
-
-
3
C
+
-
4
N
+
-
5
C
-
+
6
N
-
+
6
C
+
+
8
N
+
+
NF-κB (P50)
Oct-1
ST3dODN
IL-6

Supplement: Additional file 4 — Effect of the STAT3-decoy ODN and of IL-6 on the nuclear localization of the p50 subunit of NF-κB. Cells were either not treated (1, 2), treated with STAT3-decoy ODN (2 μg/ml) (3, 4), IL-6 (50 ng/ml) (5, 6) or both (7, 8) for 6 h. Cytoplasmic (C) and nuclear extracts (N) (see methods) were analyzed on acrylamide gels and the membranes probed with anti-p50-NF-κB and anti-Oct-1 antibodies. [file 1471-2121-12-14-S4.PPT]
